# *In vitro *fertilization and artificial activation of eggs of the direct-developing anuran *Eleutherodactylus coqui*

**DOI:** 10.1186/1477-7827-2-60

**Published:** 2004-08-05

**Authors:** Esteban Toro, Scott F Michael

**Affiliations:** 1 Department of Tropical Medicine, Box SL-17, Tulane University, New Orleans, LA 70112, USA; 2 Department of Developmental Biology, Stanford University, Stanford, CA 94045, USA

## Abstract

Although much is known about the reproductive biology of pond-breeding frogs, there is comparatively little information about terrestrial-breeding anurans, a highly successful and diverse group. This study investigates the activation and *in vitro *fertilization of eggs of the Puerto Rican coqui frog obtained by hormonally induced ovulation. We report that spontaneous activation occurs in 34% of eggs, probably in response to mechanical stress during oviposition. Artificial activation, as evidenced by the slow block to polyspermy and the onset of zygote division, was elicited both by mechanical stimulation and calcium ionophore exposure in 64% and 83% of the cases, respectively. Finally, one *in vitro *fertilization protocol showed a 27% success rate, despite the fact that about one third of all unfertilized eggs obtained by hormone injection auto-activate. We expect these findings to aid in the conservation effort of *Eleutherodactylus *frogs, the largest vertebrate genus.

## Background

The study of reproduction and its artificial manipulation is important in many fields. For example, in sea urchins, an animal's testes can be dissected and sperm is activated by exposure to seawater. Eggs can be released by injecting KCl into the perivisceral cavity, and mixing eggs and sperm *in vitro *produces fertilization, as evidenced by the appearance of the fertilization membrane and subsequent development of embryos [1]. These simple techniques have been the basis for such dissimilar studies as those of Berdishev [1], dealing with the role of fatty acids and cannabinoids in fertilization, to investigation of the gene expression patterns of hybrids by Nielsen and coworkers [2].

Artificial reproduction has also been well-studied in mammals, and cloning of eutherians from somatic cells is now common [3-8]. Harvested eggs can be enucleated and merged with a somatic cell and the reconstructed embryos cultured *in vitro *before being implanted into surrogate mothers [8]. These methods have opened up new possibilities in both basic and applied science [e.g. [9]]. Importantly, artificial fertilization has been utilized as a means of assisting with the conservation effort of declining species [10,11].

Frogs have been favorite model organisms in reproductive and developmental biology for many years, mainly because of the ease with which they can be kept in captivity; their external fertilization; easily visible development in large, transparent eggs; and large numbers and ease of manipulation of their eggs. Consequently, research on frogs has often been in the vanguard of advancement in artificial reproduction techniques, and much is known about a few model species such as the African clawed frog, *Xenopus laevis *and the North American leopard frog, *Rana pipiens *[e.g. [12-15]]. Indeed, the first vertebrate cloned from a somatic nucleus was a frog [16]. Briggs and King injected female *R. pipiens *with male pituitary glands to induce ovulation and deposition of unfertilized eggs. The eggs were mechanically activated by pricking with a needle, a process which brings the pronucleus immediately under the surface of the animal pole. Taking advantage of this situation, the pronuclei were extruded, along with a small amount of cytoplasm, using a glass needle. In other species, such as the *Xenopus *or the axolotl, UV radiation can be used to destroy the female pronucleus instead [17,18]. Development was then directed by a somatic nucleus microinjected into the cytoplasm of the enucleated egg.

In another group of experiments, Kroll and Amaya [19] developed an effective and reliable method for creating transgenic *Xenopus*: testes were macerated in solution and the sperm membranes partially dissolved, allowing access to the condensed chromosomes. Linearized bacterial plasmids containing genes of interest were mixed in with the sperm solution and recombinant ligase was used to covalently insert the bacterial plasmids into the sperm genomic DNA, resulting in the insertion of many copies of the plasmid construct into each genome. These nuclei were then microinjected into mature eggs, generating, under appropriate conditions, hundreds of nonmosaic, transgenic embryos. Such techniques allow the investigation of gene function in these species [e.g. [20]].

Clearly, there are enormous advantages to being able to manipulate a species' reproduction in the laboratory. However, despite the multiplicity of studies concentrating on anurans, to date all model species are aquatic breeders. Yet amphibians have the largest diversity of breeding strategies among terrestrial vertebrates, and it is to be expected that species with different reproductive strategies will require different methods for their manipulation in the laboratory. Therefore, many species remain experimentally intractable. Notably, terrestrial-breeding frogs, a very large and diverse group of organisms, are largely inaccessible to reproductive investigations.

The neotropical frog genus *Eleutherodactylus *is characterized by terrestrial breeding and direct development without an aquatic larval stage. With more than seven hundred described species, this is the largest vertebrate genus [21,22]. There has been considerable experimental attention focused on *Eleutherodactylus *frogs, ranging from basic developmental biology [23-26]; to ecology [e.g. [27-30]]; to the evolution of development [23,31]. However, there are as yet no available techniques for performing *in vitro *fertilization in these frogs. The development of such techniques would allow additional investigations into the genetic regulation of direct development in these species and would also assist with conservation of declining populations, an important goal considering the fact that many species of *Eleutherodactylus *are declining, and several are already extinct [32,33].

*Eleutherodactylus coqui *are small tree frogs with internal fertilization and direct development [34]. This species is extremely common in the forests of Puerto Rico, and it has been found that their population size is limited by the availability of retreat sites, as opposed to food resources [35]. As with all other studied *Eleutherodactylus *species, *E. coqui *embryos develop directly into tiny froglets in terrestrial eggs, without a tadpole stage [36]. Protocols for the husbandry of these frogs have been reported, and it is possible to maintain them in the laboratory for multiple generations [37,38]. A method has also been developed to induce ovulation using an artificial form of luteinizing hormone-releasing hormone (LHRH) [39]. It is know in this species that sperm entry occurs at a small disc at the animal pole of the egg, and that polyspermy is apparently common but does not interfere with development [40]. Cortical granules and their exocitosis have also been described using electron microscopy [40], but the large (5 mm diameter), opaque and featureless eggs make it difficult to observe the rising of a fertilization membrane. As *E. coqui *is arguably the best-studied terrestrial-breeding frog, we have focused on this particular species as a model for the development of reproductive techniques.

## Materials and Methods

Adult *Eleutherodactylus coqui *frogs were collected in Puerto Rico near El Verde Field Station in the Luquillo mountains and transported to Tulane University where they were housed and fed as previously described [37]. All animals were handled and experiments performed in accordance with the standards outlined in the NIH Guide for the Care and Use of Laboratory Animals.

Natural matings were performed by placing a gravid female and a calling male together as described [37]. Mature, unfertilized eggs were obtained by injection of gravid females with 20 μg of des-Gly, D-Ala LHRH ethylamide (Sigma, St. Louis, MO. Catalog Number: L4513), as reported [39]. Hormonally induced females were placed in plastic bags and allowed to deposit unfertilized eggs. Eggs were experimentally manipulated without moving them from the surface of the plastic bag where they were deposited. Sperm was obtained from adult male frogs that were anesthetized by immersion in 5% benzocaine solution, decapitated and double-pithed. Testes were removed by dissection and macerated with fine forceps.

*In vitro *fertilization (IVF) experiments using sperm in solution were carried out by macerating the testes from a single frog in 500 μL of sperm dilution buffer (SDB: 10 mM NaCl, 0.2 mM KCl, 0.1 mM CaCl_2_, 0.1 mM MgCl_2_, 0.5 mM Hepes pH 7.5), and adding this dropwise over the tops of the eggs or injecting it under the jelly coat using a tuberculin syringe (28.5 gauge, 13 mm length). Alternatively, small pieces of macerated testes were placed directly on top of each egg without the use of buffer solution. Incubation of sperm with egg jelly was accomplished by vigorously vortexing the jelly from one egg in 100 μl of SDB. Sperm was then incubated for 10 min in the jelly/buffer supernatant.

Sperm preparations were checked for morphology, movement and membrane integrity by fluorescent microscopy with Live/Dead sperm stain (propidium iodide and SYBR 14) (Molecular Probes, Eugene, OR) using an Olympus BH-2 microscope [41]. Additionally, sperm were stained with 1 μM Lysosensor green fluorescent dye (Molecular Probes, Eugene, OR) and imaged with a Zeiss LSM 510 META laser-scanning confocal microscope [42].

**Figure 1:**
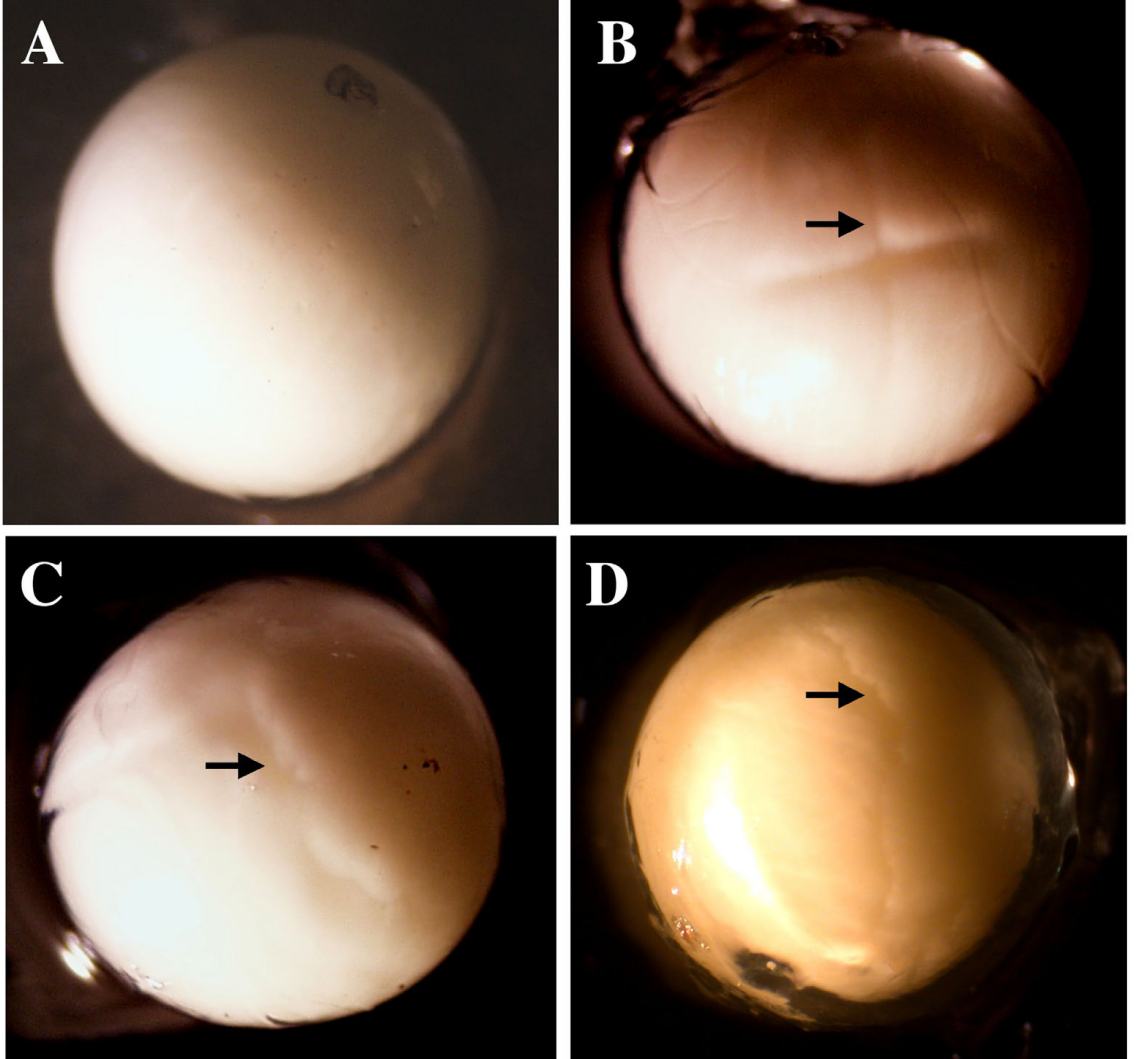
Early egg development. **A) **Untouched, unfertilized egg. The surface of the egg under the jelly coat is featureless. **B) **Sperm-activated egg at six hours post-fertilization at the four-cell stage. Note the straight, ordered cleavage pattern (arrow). **C) **Artificially activated egg pseudocleaving. Note the jagged and disorganized cleavage pattern (arrow). **D) **An egg pseudocleaving at 16 hours. This egg was not handled and did not cleave within the first ten hours after deposition. At ten hours it was poked, and started pseudocleavage shortly thereafter (arrow). Scale bar = 1 mm.

For artificial activation experiments, mature eggs were either mechanically stimulated by gentle poking with fine forceps, taking care to penetrate the jelly coat but not the plasma membrane, or immersed in a solution containing 10 mM CaCl_2 _with 0.1 mM A23187 calcium ionophore. Scoring of activation was done by noting the appearance of the first cleavage furrow (see figure 1). Artificial activation experiments were also performed on oocytes dissected directly from the ovisac of gravid females. These oocytes were similarly treated by poking or exposure to 10 mM CaCl_2 _with 0.1 mM A23187 calcium ionophore, with or without pretreatment for 12 hrs. with 3 μM progesterone [43].

Eggs were allowed to develop at room temperature in parafilm-sealed 60 or 100 mm polystyrene petri dishes and were moistened with an antibiotic solution consisting of 25 μg/ml amphotericin B, 10 U/ml penicillin and 10 ug/ml streptomycin. Eggs were scored as successfully fertilized following neurulation (stage 2 *sensu *Townsend and Stewart [36]; stage 14 *sensu *Gosner [44]).

## Results and Discussion

### Artificial activation

Table 1 shows the activation effects of either mechanical stimulation or A23187 calcium ionophore exposure on the unfertilized eggs of *E. coqui*. This ionophore non-specifically activates the unfertilized eggs of a variety of species through stimulation of a calcium-dependent signalling cascade [45]. Ten hours after laying, 11 of 32 eggs (34%) pseudocleaved, even if left undisturbed. As unfertilized eggs do not posses a centrosome, if they are artificially activated, the mitotic apparatus cannot form properly and divisions are irregular. This well-described process is called pseudocleavage [[46]; figure 1]). Activation almost doubled to 28 of 44 (64%) when the eggs were poked with forceps. Further, 30 of 36 eggs (83%) exposed to 0.1 mM calcium ionophore pseudocleaved. These percentages include both eggs that would have auto-activated –presumably 34%- as well as eggs that were activated by the mechanical or chemical treatments.

**Table 1:** Artificial activation of *E. coqui *eggs

**Egg origin**	**Treatment**	**Number cleaving at 10 hours/Number tested (%)**	**Number cleaving at 16 hours, after being poked at 10 hours/Number tested (%)**
Eggs laid in response to hormone treatment	Undisturbed	11/32 (34)	6/8 (75)
	Poked	28/44 (64)	-
	A23187	30/36 (83)	-
Oocytes dissected directly from ovisac	Undisturbed	0/40	-
	A23187	0/40	-
	Progesterone	0/40	-
	Progesterone & AA23187	0/40	-

To test whether the pseudocleavage response was an effect of our stimuli and not a reaction tied to other uncontrolled variables, we examined eight eggs that had remained undisturbed and that had not started pseudocleavage ten hours after laying. At this time, we poked them with fine forceps, and 6 of 8 (75%) began pseudocleaving six hours later (table 1). This delay in activation as a response to a delay in the stimulus is a strong indication that our manipulation is in fact responsible for eliciting the onset of cell division.

An interesting observation was that one third of all eggs deposited in response to hormone treatment activated of their own accord. This may be due to the mechanical stress to which the eggs are exposed during oviposition. Clearly, mechanical stimuli are able to activate the eggs, and stress incurred in during transit from the ovisac may be sufficient to cause activation. This would presumably not affect *E. coqui *during natural matings because this species undergoes internal fertilization, and the eggs will have already been fertilized prior to deposition [34]. In order to examine this hypothesis, we dissected oocytes directly from a female's ovisac, circumventing the passage through the oviduct and cloaca, and attempted to activate them with calcium ionophore (table 1). Controls were also performed with and without progesterone pretreatment in order to induce maturation. Since there is no information on the stage at which *E. coqui *eggs are arrested or what signal takes them out of their arrest, we followed procedures used in *Xenopus *[43]. However, none of these oocytes activated, regardless of the treatment. This may be because the oocytes did not respond to treatment with progesterone, and so never matured. Another possibility is that oocytes need to receive a signal from the oviducts and/or be coated in jelly before they can mature.

**Figure 2:**
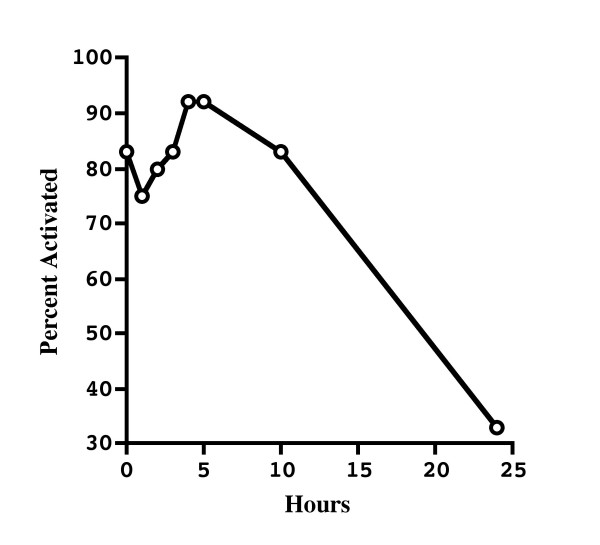
Artificial activation of *E. coqui *eggs in relation to time after deposition. Unfertilized eggs were treated with calcium ionophore to induce activation at 0 (n = 12), 1 (n = 12), 2 (n = 10), 3 (n = 12), 4 (n = 12), 5 (n = 12), 10 (n = 18), and 24 (n = 18) hours. Eggs were scored for activation by the presence of cleavage furrows at 6 hours post treatment. Note that for the 10 and 24 hour time points, six eggs at each time point had already auto-activated by 6 hours post deposition. At the 10 hour time point, nine additional eggs activated later in response to ionophore treatment, while at 24 hours, no additional eggs were observed to activate after ionophore treatment.

The ability to initiate activation after a long delay was interesting as we suspected that *E. coqui *eggs might be sensitive to aging, as has been reported under certain conditions for the externally fertilizing *X. laevis *[47]. Because *E. coqui *has internal fertilization, we suspected that eggs laid unfertilized might degenerate rapidly, complicating the artificial manipulation of this species' reproduction. Consequently, we investigated the ability of eggs to be activated as a function of time. When we artificially activated eggs with the calcium ionophore at different time points and examined them six hours post treatment, the percentage that pseudocleaved was high even ten hours after being laid (Figure 2). Twenty-four hours after deposition, however, the eggs were no longer able to activate. Thus, there is an extended period in which it is possible to carry out experiments without concern for a decrease in activation potential.

#### In vitro fertilization

The average fertilization efficiency for natural matings conducted in our laboratory was 72% (see table 2). As roughly one third of all unfertilized eggs laid in response to hormone treatment auto-activate (table 1), and so only approximately 66% of the eggs in a given clutch will actually be receptive to sperm. If we assume these two factors to be independent -because we hypothesize that the eggs auto-activate during laying, a problem that doesn't arise in natural matings- then only 48 of every hundred eggs will be available for IVF (100*0.66*0.72). However, despite these complications, we were able to obtain an *in vitro *fertilization efficiency of 27% -or, rather, 56% of all receptive eggs (27/0.48) – by simply mincing the testes and adding them directly over the eggs (see table 2). Other IVF techniques were not as successful. Using sperm diluted in SDB resulted in only a 12% total fertilization efficiency (table 3).

**Table 2:** Natural mating fertilization percentages for *E. coqui *in captivity.

**Clutch**	**Number of eggs laid**	**Number that developed to neurula (%)**
1	39	24 (62)
2	58	36 (62)
3	30	28 (93)
4	42	34 (81)
**Total**	169	122 (72)

**Table 3:** *In vitro *fertilization of *E. coqui *eggs

**Fertilization protocol**	**Number that developed to neurula/Number tested (%)**
Sperm solution dripped over the eggs	**5/63 (8)**
Testes minced directly over eggs	**8/30 (27)**
Sperm solution injected under the jelly coat	**0/12**
Sperm incubated in jelly buffer and then injected under the jelly coat	**1/10 (10)**
Poked, then fertilized 15 minutes later by mincing testes directly over the eggs	**0/36**

Sperm concentration may play a role in fertilization efficiency as the use of diluted sperm resulted in decreased fertilization. In support of this possibility, polyspermy has been observed in this species and is apparently not deleterious to fertilization and development [40]. A second possibility is that fertilization efficiency is linked to sperm capacitation and acrosome reaction. We attempted to study this possibility by examining the acrosomes of fresh and treated sperm using Lysosensor green fluorescent dye (Molecular Probes, Eugene, OR). This dye concentrates in low pH vesicles of living cells through an unknown mechanism and was shown to accumulate and preferentially stain the acrosome in *X. laevis *sperm [42]. However, we were unable to observe acrosome-specific staining in *E. coqui *sperm using this dye (data not shown). We also examined the possibility that a component of the egg jelly coat may be important for sperm capacitance. To test this, we incubated sperm with SDB and jelly, or SDB alone, and injected this under the jelly coat of eggs. None of 12 eggs were fertilized by sperm incubated with SDB alone, while pre-incubation of sperm with an extract of the jelly coat in SDB resulted in one fertilization out of 10 eggs (10%). This is considerably less than the 27% efficiency following direct placement of minced testes over the eggs, but suggests that interactions between sperm and the jelly coat may play a role in sperm capacitance and subsequent fertilization.

To test the functional response of the eggs, we attempted to fertilize artificially activated eggs. As was explained above, we were able to achieve an IVF success rate of 27%. However, if we poked the eggs fifteen minutes prior to direct fertilization, none (0/36) developed (see table 3). If we assume our expected fertilization rate to be 25%, the possibility of this result being due to chance is (1-0.25)^36 ^= 3.2 × 10^-5^, or less than one in ten thousand. This shows that fifteen minutes after being poked the eggs have established a block to polyspermy, one of the defining functional characteristics of activation. Hence, although the first visible indication of activation –the formation of the first cleavage furrow- will not be seen for six hours, we can conclude that the egg is undergoing the normal activation processes within minutes of being stimulated.

## Conclusions

In an effort to conserve declining populations of animals, the development of protocols for the artificial manipulation of reproduction is of great interest. In the case of the neotropical frog *E. coqui*, we have observed that a large proportion of eggs that are laid unfertilized auto-activate. We showed that *E. coqui *eggs are easily activated by mechanical stimuli, leading to a need for careful manipulation of unfertilized eggs in all reproduction studies. Further, the cleavage pattern seen in mechanically activated eggs is similar to that of both auto-activated and chemically activated eggs, suggesting that mechanical stress, probably incurred in during oviposition, is responsible for the auto-activation mentioned above (table 1). Facile auto-activation of eggs has been reported in other species, complicating reproductive manipulation [48]. In *E. coqui*, this phenomenon may relate to internal fertilization, and it would be interesting to investigate auto-activation of unfertilized eggs in closely related, externally fertilizing species such as *E. antillensis *[38]. Some *E. coqui *eggs, however, remain intact and can be manipulated, showing signs of activation both at the morphological level, through the initiation of development, as well as the functional, through the slow block to polyspermy. By careful handling, we are now able to fertilize over half of the remaining, functionally viable eggs using a simple procedure. Finally, we have shown that *E. coqui *eggs do not degenerate rapidly and are capable of undergoing activation up to ten hours after deposition, thus creating a window of time for carrying out experimental procedures. Our results demonstrate efficient IVF in an internally fertilizing, terrestrial-breeding frog and help lay the foundation for future research and conservation possibilities in this unusually large genus of amphibians.

## Authors' contributions

ET carried out the experiments and prepared the manuscript. SFM carried out preliminary experiments and supervised the manuscript. The study was conceived jointly. Both authors approved the final manuscript.
